# Socioeconomic differences in primary healthcare use before childhood cancer diagnosis: a register-based cohort study in Sweden

**DOI:** 10.1136/bmjpo-2026-005059

**Published:** 2026-09-24

**Authors:** Julia Reißinger, Christina-Evmorfia Kampitsi, Maria Feychting, Hanna Mogensen, Marios Rossides, Eric Chen, Arja Harila, Kleopatra Georgantzi, Giorgio Tettamanti, Javier Louro

**Affiliations:** 1 Unit of Epidemiology, Institute of Environmental Medicine, Karolinska Institutet, Stockholm, Sweden; 2 Department of Immunology, Genetics and Pathology, Cancer Precision Medicine, Uppsala University, Uppsala, Sweden; 3 Department of Respiratory Medicine and Allergy, Theme Inflammation and Ageing, Karolinska University Hospital, Stockholm, Sweden; 4 Academic Primary Health Care Centre, Region Stockholm, Stockholm, Sweden; 5 Division of Family Medicine and Primary Care, Department of Neurobiology, Care Sciences and Society, Karolinska Institutet, Huddinge, Sweden; 6 Department of Women’s and Children’s Health, Uppsala University Hospital, Uppsala, Sweden; 7 Childhood Cancer Research Unit, Department of Women’s and Children’s Health, Karolinska Institutet, Stockholm, Sweden; 8 Division of Paediatric Haematology-Oncology, Astrid Lindgren Children's Hospital, Karolinska University Hospital, Stockholm, Sweden; 9 Department of Molecular Medicine and Surgery, Karolinska Institutet, Stockholm, Sweden

**Keywords:** Child, Epidemiology

## Abstract

**Background:**

Early diagnosis of childhood cancer remains challenging because initial symptoms are common, nonspecific and have low predictive value in primary healthcare (PHC). We assessed whether socioeconomic differences shape pre-diagnostic PHC pathways before childhood cancer diagnosis.

**Methods:**

We conducted a register-based matched cohort study using PHC data from three Swedish regions between 2001 and 2022, covering 54% of the general paediatric population in Sweden. Children aged 0–19 years with a first cancer diagnosis were matched 1:25 to cancer-free population references by age, sex and region. In-person PHC physician contacts in the year before the index date were analysed. Logistic and negative binomial regression models were used to assess socioeconomic differences in PHC utilisation and PHC contact frequency, respectively stratified by diagnostic group.

**Results:**

The study included 3711 childhood cancer patients and 93 181 references. Children with cancer had more PHC contacts than population references in the year before diagnosis (mean 2.28 vs 1.27, mean difference 1.01, 95% CI 0.94 to 1.08; p<0.001), although 22%–49% had no PHC contact 3 months before diagnosis depending on diagnostic group. Maternal educational gradients in PHC use were present in the general paediatric population but largely absent among children with cancer. An exception was central nervous system (CNS) tumours, for which children of mothers with low educational attainment had 28% more prediagnostic PHC contacts than those with highly educated mothers.

**Conclusion:**

Socioeconomic differences in PHC utilisation by maternal educational level largely diminish as children approach a cancer diagnosis, except for CNS tumours, where increased contacts among children of mothers with low educational attainment may indicate a prolonged or delayed diagnosis.

**WHAT IS ALREADY KNOWN ON THIS TOPIC**

**WHAT THIS STUDY ADDS**

- While children with cancer had more primary care visits than their peers, especially in the 3 months before diagnosis, these consultation patterns did not vary by maternal educational level, unlike in the general paediatric population. The only exception was central nervous system (CNS) tumours, where children of mothers with lower educational attainment experienced more consultations before diagnosis.

**HOW THIS STUDY MIGHT AFFECT RESEARCH, PRACTICE OR POLICY**

- Primary care visit frequency is unlikely to explain socioeconomic survival disparities for most paediatric cancers. Nevertheless, the longer diagnostic route observed for CNS tumours warrants targeted clinical strategies to improve referral pathways for disadvantaged families.

## Introduction

 Childhood cancer is the leading cause of disease-related mortality among children in high-income countries.^1^ Early symptoms are often nonspecific, including fatigue, fever, nausea or vomiting, which are more commonly associated with less severe diseases.^2^ As a result, childhood cancer alert symptoms listed in clinical guidelines have low positive predictive value for diagnosing cancer.^3^ Consequently, identifying children with cancer and referring them to specialised care presents a challenge to primary healthcare (PHC) providers, who have to weigh the negative economic and psychological implications of over-testing children against the risk of failing to identify a malignancy.^4^

The heterogeneity of symptom presentation and the variability in diagnostic pathways, influenced by childhood diagnostic group and patient characteristics, further complicate timely diagnosis.^5^ The literature concerning the pathway that childhood cancer patients follow before diagnosis is limited. In the Swedish context, the only available bibliography derives from a small-scale study conducted in Skåne based on data collected between 1984 and 1995 and encompassing 68 paediatric patients.^6^ This study found that 68% of children with cancer initially sought care in PHC, with the remainder presenting directly to hospital settings, and highlighted potential delays both in healthcare seeking and in diagnosis.

These delays in diagnosis, driven by diagnostic complexity, may have profound implications for patients and their families and have been associated with poorer prognosis for certain childhood diagnostic groups.^7 8^ Timely diagnosis is critical, as it enables treatment at an earlier stage, a key prognostic factor for childhood cancer survival.^9^ In addition, delayed diagnosis may lead to avoidable suffering through prolonged symptomatic periods and can increase the risk of permanent complications by allowing tumour progression, necessitating more intensive therapy and raising the likelihood of long-term organ, neurocognitive or endocrine damage.^4^

Socioeconomic differences in childhood cancer survival have been repeatedly observed, with worse outcomes for children from families of lower socioeconomic status (SES).^10^ These differences may reflect disparities in the incidence of cancer subtypes with poorer prognosis, disease severity at diagnosis or treatment adherence.^11^ Children from lower SES families might experience delayed childhood cancer diagnosis as a result of differences in parental health literacy as well as social and cultural capital, influencing the ability of parents to interact with healthcare providers prior to diagnosis.^12^

Previous research regarding the relationship between SES and diagnostic delays in childhood cancer from high-income countries with healthcare systems similar to the Swedish have provided conflicting findings. Studies found no SES-related differences in the interval from symptom onset to childhood cancer diagnosis^13^ or from the first symptomatic PHC visit to diagnosis.^14 15^ In contrast, studies focusing on the frequency of PHC contacts before diagnosis, a different indicator for timeliness of diagnosis, have identified SES disparities.^12 16^ These studies found that children with cancer from lower SES backgrounds were more likely to have had frequent prediagnostic contact with the healthcare system and required more additional consultations before their diagnosis compared with children from higher SES backgrounds.^12 16^

Using register-based PHC data, we examined whether childhood cancer patients showed SES differences in PHC contact frequency during the year before diagnosis. To determine whether any SES patterns were specific to paediatric cancer or reflected general care-seeking behaviour, we also compared pre-diagnostic PHC use among childhood cancer patients with that of an age- and sex-matched Swedish paediatric population.

## Methods

### Setting

The Swedish healthcare system is tax-funded and provides universal coverage to all residents. For children younger than 18 years old, healthcare at all levels is free of cost.^17^ PHC is responsible for addressing common healthcare needs in the population, providing easily accessible care and implementing preventive measures. Access to specialist services is regulated through regional referral pathways, with PHC functioning as the principal gatekeeper and typically serving as patients’ first point of contact with the healthcare system.

### Study population

We performed a register-based matched cohort study based on data from the PHC registers in three Swedish regions, Stockholm, Västra Götaland and Skåne, which together account for 54.4% of the Swedish population aged 0 to 19 years. All Swedish citizens as well as immigrants with the right to stay for more than 1 year are assigned a unique personal identification number, which allowed us to link regional PHC data with other national registers.^18^

Childhood cancer patients were defined as individuals receiving their first cancer diagnosis at 0 to 19 years of age, identified through the National Cancer Register.^19^ For this study, childhood cancer diagnoses were retrieved from 1 January 2001 (Västra Götaland), 1 January 2003 (Skåne) and 1 January 2008 (Stockholm) until 31 December 2022.

For each childhood cancer case, 25 population references without any prior cancer diagnosis at the case’s diagnosis date (index date) were randomly selected from the Total Population Register,^20^ using incidence density sampling and matched on sex, region of residence at the index date and year and month of birth. The index date for population references was defined as the date of cancer diagnosis of their matched case.

### Childhood cancer classification

Childhood cancer patients were stratified based on the diagnosis registered in the National Cancer Register, according to the International Classification of Childhood Cancer, Third Edition (ICCC-3). Five diagnostic groups were defined: (1) acute lymphoid leukaemia (ALL, ICCC-3 group Ia), (2) acute myeloid leukaemia (AML, ICCC-3 group Ib), (3) lymphomas (ICCC-3 group II), (4) central nervous system tumours (CNS, ICCC-3 group III) and (5) other non-CNS solid tumours (ICCC 3 groups IV-XII). Cases with leukaemia subtypes other than ALL and AML were excluded due to small sample sizes and insufficient statistical power. Additionally, diagnoses that could not be classified under ICCC-3 were not included.

### Primary healthcare contacts

For each PHC contact, we obtained information on the type of contact (in-person, telephone or correspondence) and the healthcare professional involved. To ensure relevance to the cancer diagnosis, only in-person visits to PHC physicians occurring within the year prior to the childhood cancer diagnosis or corresponding index date were included in the analysis. Data were extracted from the regional healthcare databases VAL in Stockholm, VEGA in Västra Götaland and KVB in Skåne. Contacts were divided into 3-month intervals: (1) 12 to 9 months, (2) 9 to 6 months, (3) 6 to 3 months and (4) 3 to 0 months before cancer diagnosis or index date.

### Maternal socioeconomic status and country of birth

Parents of the childhood cancer patients and population references were identified through the Multigeneration Register.^21^ Maternal education was used to define SES, as it has been shown that low maternal education is associated with poorer childhood cancer prognosis.^22^ Maternal disposable income was included as the SES indicator in a secondary analysis. Both indicators were extracted from the Swedish longitudinal integrated database for health insurance and labour market studies (LISA)^23^ for the calendar year preceding childhood cancer diagnosis/index date. Education was the primary SES indicator given its well-established relation with childhood cancer survival early after diagnosis.^11^ It was categorised based on the highest attained level of education into: (1) low (compulsory education or less), (2) intermediate (2–3 years of upper secondary education) or (3) high (university education and higher). Maternal disposable income was categorised into quintiles based on the yearly income distribution among mothers of the population references. Maternal country of birth was included as a confounding variable, as it may be associated both with PHC utilisation, due to potential language barriers and adaptation to the healthcare system, and with maternal SES. Maternal country of birth was categorised as either European Union (EU) or non-EU.

### Statistical analysis

The mean number of PHC contacts for cases and population references during the year before childhood cancer diagnosis/index date was calculated overall and stratified by diagnostic group and demographic and socioeconomic variables.

For both population references and childhood cancer patients, we used standard logistic regression to estimate adjusted ORs (aORs) and 95% CIs for having no PHC contacts within the 3 months preceding the index date across levels of SES and stratified by diagnostic group.

We estimated the additional number of PHC contacts attributable to the childhood cancer diagnosis by subtracting, for each case, the average number of contacts among their matched population references, representing the cancer-free population of the same age, sex and region. This calculation was performed for each of the four quarters within the year preceding the diagnosis and visualised using bar plots with 95% CIs, calculated using the normal distribution.

To analyse the number of PHC contacts in the 3 months period before the index date, we used negative binomial regression models to estimate adjusted incidence rate ratios (aIRRs) and 95% CIs across levels of SES for both population references and childhood cancer patients stratified by diagnostic group. This model was selected to account for the non-normal, right-skewed distribution and overdispersion of the contact count data. Only individuals with at least one PHC contact during this period were included to ensure that the information captured by the previous analysis was not reflected in both analyses. A secondary analysis was conducted using maternal income instead of maternal education, and sensitivity analyses extended the time window to 6 months, 9 months and 1 year preceding the index date.

All analyses were stratified by diagnostic group, and all models were adjusted for calendar year of cancer diagnosis/index date, sex, region, maternal country of birth and maternal age at diagnosis. Analyses were stratified by case-population reference status to assess socioeconomic gradients within each cohort independently. Matched population references served to establish population-level baseline reference patterns rather than to conduct formal effect-modification testing. As interaction terms were not included in the main models, relative differences in effect magnitude between cases and controls are presented for contextual baseline comparison. All analyses were performed using complete cases. All analyses were performed in R V.4.3.1.^24^

The Strengthening the Reporting of Observational Studies in Epidemiology (STROBE) guidelines^25^ were used when designing the study and preparing the manuscript. Patients or the public were not involved in the design, or conduct, or reporting, or dissemination plans of our research.

## Results

### Study population characteristics and average number of PHC contacts

In total, 3711 childhood cancer patients and 93 181 population references were included in the study (table 1). Childhood cancer patients had a higher mean number of PHC contacts in the year before diagnosis compared with their population references (2.28 vs 1.27 contacts, respectively). The mean number of PHC contacts was highest for children later diagnosed with ALL (2.74) and lowest for those with CNS tumours (1.98). For both the population reference group and childhood cancer patients, the highest number of contacts was observed in the youngest age group (0–4 years), reflecting the routine early childhood check-ups performed at children’s health centres. PHC contacts decreased across all SES levels in 2021 during the COVID-19 pandemic, while contacts for children diagnosed in 2022 were comparable to pre-pandemic values.

**Table 1:** Characteristics of the study population

		Childhood cancer cases		Population references	
		Total number (%)	Mean number of contacts 0–12 months before index date (SD)	Total number (%)	Mean number of contacts 0–12 months before index date (SD)
Total		3711	2.28 (2.12)	93 181	1.27 (1.76)
Sex					
	Male	1960 (52.8)	2.20 (2.07)	49 078 (52.7)	1.24 (1.77)
	Female	1751 (47.2)	2.37 (2.17)	44 103 (47.3)	1.31 (1.76)
Age at diagnosis/index (years)					
	0–4	1075 (29.0)	3.04 (2.57)	27 016 (29.0)	2.10 (2.24)
	5–9	737 (19.9)	1.79 (1.69)	18 387 (19.7)	0.78 (1.20)
	10–14	1227 (33.1)	2.03 (1.88)	30 955 (33.2)	0.99 (1.47)
	15–19	672 (18.1)	2.06 (1.84)	16 823 (18.1)	1.00 (1.43)
Region					
	Skåne	996 (26.8)	1.98 (1.86)	25 016 (26.8)	1.09 (1.63)
	Stockholm	1293 (34.8)	2.46 (2.26)	32 470 (34.8)	1.46 (1.91)
	Västra Götalands	1422 (38.3)	2.33 (2.14)	35 695 (38.3)	1.23 (1.69)
Diagnostic group					
	ALL	729 (19.6)	2.74 (2.34)	18 124 (19.5)	1.49 (1.94)
	AML	131 (3.5)	2.27 (2.24)	3271 (3.5)	1.43 (1.88)
	Lymphomas	1027 (27.7)	2.26 (1.72)	25 694 (27.6)	0.96 (1.44)
	CNS	462 (12.4)	1.98 (2.03)	11 598 (12.4)	1.19 (1.68)
	Non-CNS solid tumours	1362 (36.7)	2.27 (2.14)	34 494 (37.0)	1.30 (1.80)
Calendar period of index date					
	(–2010)	993 (26.8)	1.98 (2.06)	24 907 (26.7)	1.17 (1.74)
	(2010–2014)	853 (23.0)	2.64 (2.29)	21 401 (23.0)	1.54 (1.97)
	(2014–2018)	801 (21.6)	2.38 (2.15)	20 117 (21.6)	1.35 (1.76)
	(2018–)	1064 (28.7)	2.19 (1.97)	26 756 (28.7)	1.09 (1.57)
Maternal country of birth					
	EU	2869 (77.3)	2.21 (2.09)	71 187 (76.4)	1.24 (1.72)
	Outside EU	805 (21.7)	2.54 (2.24)	20 627 (22.1)	1.39 (1.93)
	Missing	37 (1.0)	1.84 (1.62)	1367 (1.5)	0.89 (1.43)
Maternal education					
	High	1810 (48.8)	2.27 (2.10)	43 234 (46.4)	1.24 (1.70)
	Intermediate	1417 (38.2)	2.25 (2.13)	36 232 (38.9)	1.30 (1.78)
	Low	394 (10.6)	2.37 (2.08)	10 765 (11.6)	1.34 (1.91)
	Missing	90 (2.4)	2.52 (2.64)	2950 (3.2)	1.10 (1.79)
Maternal income					
	Q1 – Highest	920 (24.8)	1.94 (1.82)	21 497 (23.1)	1.05 (1.51)
	Q2	724 (19.5)	2.29 (2.05)	17 916 (19.2)	1.13 (1.61)
	Q3	606 (16.3)	2.22 (2.08)	16 423 (17.6)	1.24 (1.71)
	Q4	671 (18.1)	2.41 (2.22)	16 763 (18.0)	1.46 (1.92)
	Q5 – Lowest	727 (19.6)	2.66 (2.43)	18 589 (19.9)	1.56 (2.02)
	Missing	63 (1.7)	1.94 (1.69)	1993 (2.10)	0.87 (1.41)

ALL, Acute Lymphoid Leukaemia; AML, Acute Myeloid Leukaemia; CNS, Central Nervous System; EU, European Union; Q1–Q5, Quintiles 1–5.

### Utilisation of PHC three months before diagnosis

Absolute numbers and proportions of individuals with at least one PHC contact across categories are detailed in figure 1. Among population references, children of mothers with low or intermediate educational backgrounds had higher odds of having at least one PHC contact within 3 months before the index date compared with children of highly educated mothers (aOR_low_=1.12; 95% CI 1.06 to 1.18 and aOR_intermediate_=1.13; 95% CI 1.09 to 1.17) (figure 1), reflecting lower PHC use in higher socioeconomic groups. Among childhood cancer patients, this pattern was largely absent. An exception was observed for non-CNS solid tumours, where children of mothers with low-education were more likely to have at least one PHC contact before cancer diagnosis than those of highly educated mothers (aOR_low_=1.51; 95% CI 1.02 to 2.26). However, for AML, the opposite association was found, with children from low-education families having lower odds of having at least one PHC contact during the 3 months before diagnosis compared with those of highly educated mothers (aOR_low_=0.20; 95% CI 0.05 to 0.66). It should be noted that this diagnostic group included the smallest number of cases, reflected by the wide CI, and these results should therefore be interpreted with caution. For maternal income, similarly to for education, there was an inverse gradient among population references related to the odds of having at least one PHC consultation in the 3 months prior to the index date. In contrast, no clear differences were observed among childhood cancer patients (online supplemental figure 1).

**Figure 1:**
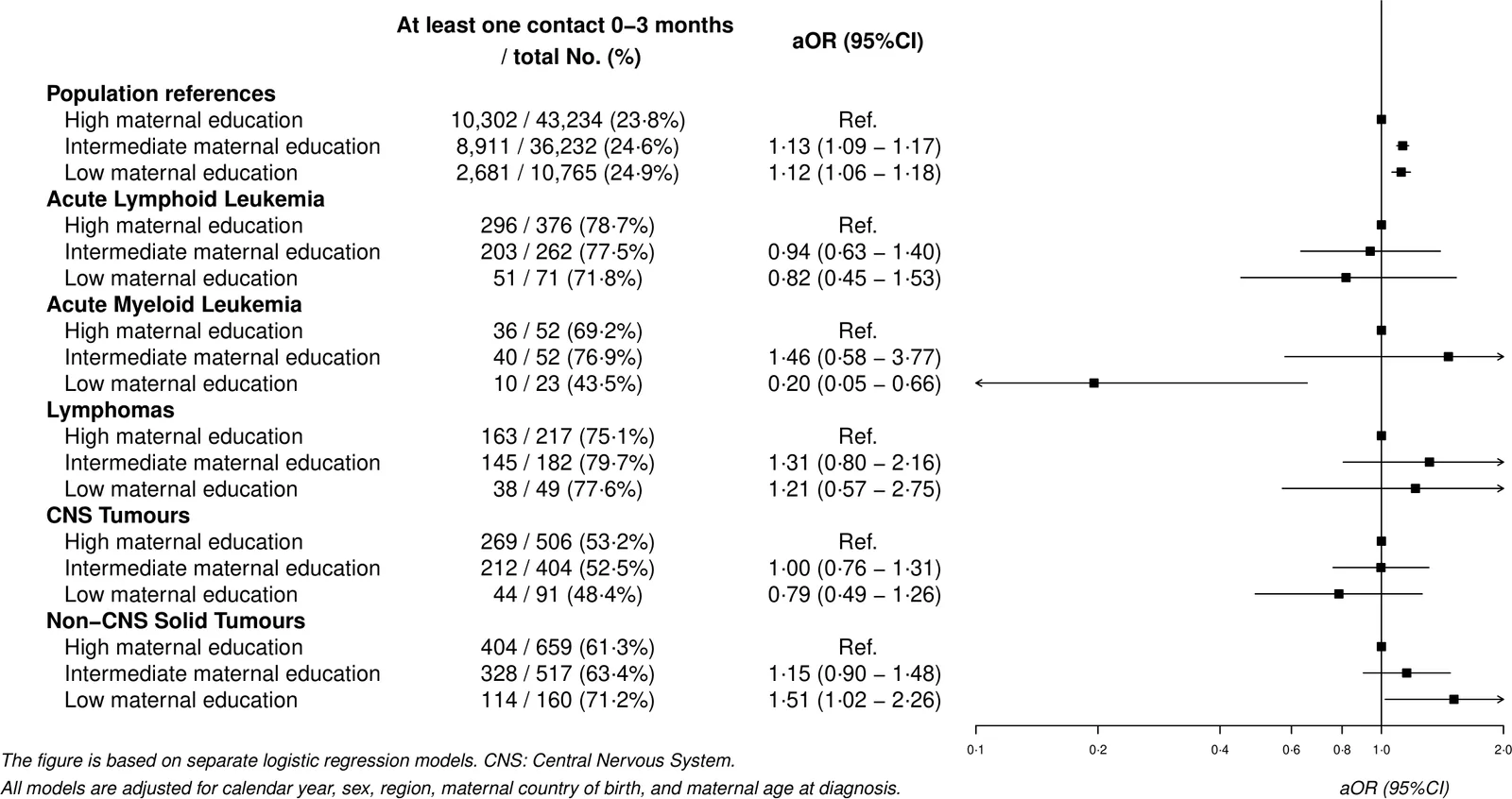
Adjusted ORs (aOR) of no primary healthcare contacts in the 3 months before diagnosis or index date across levels of maternal education, stratified by diagnostic group.

### Additional PHC contacts attributable to childhood cancer

For all diagnostic groups, the highest number of additional PHC contacts comparing childhood cancer patients with the reference population was observed during the last 3 months before childhood cancer diagnosis (figure 2); however, patterns varied by diagnostic group. For lymphomas, an increase in the number of additional contacts was evident from 6 months before diagnosis, whereas for solid tumours (both CNS and non-CNS), this increase became apparent 9 months before diagnosis.

**Figure 2:**
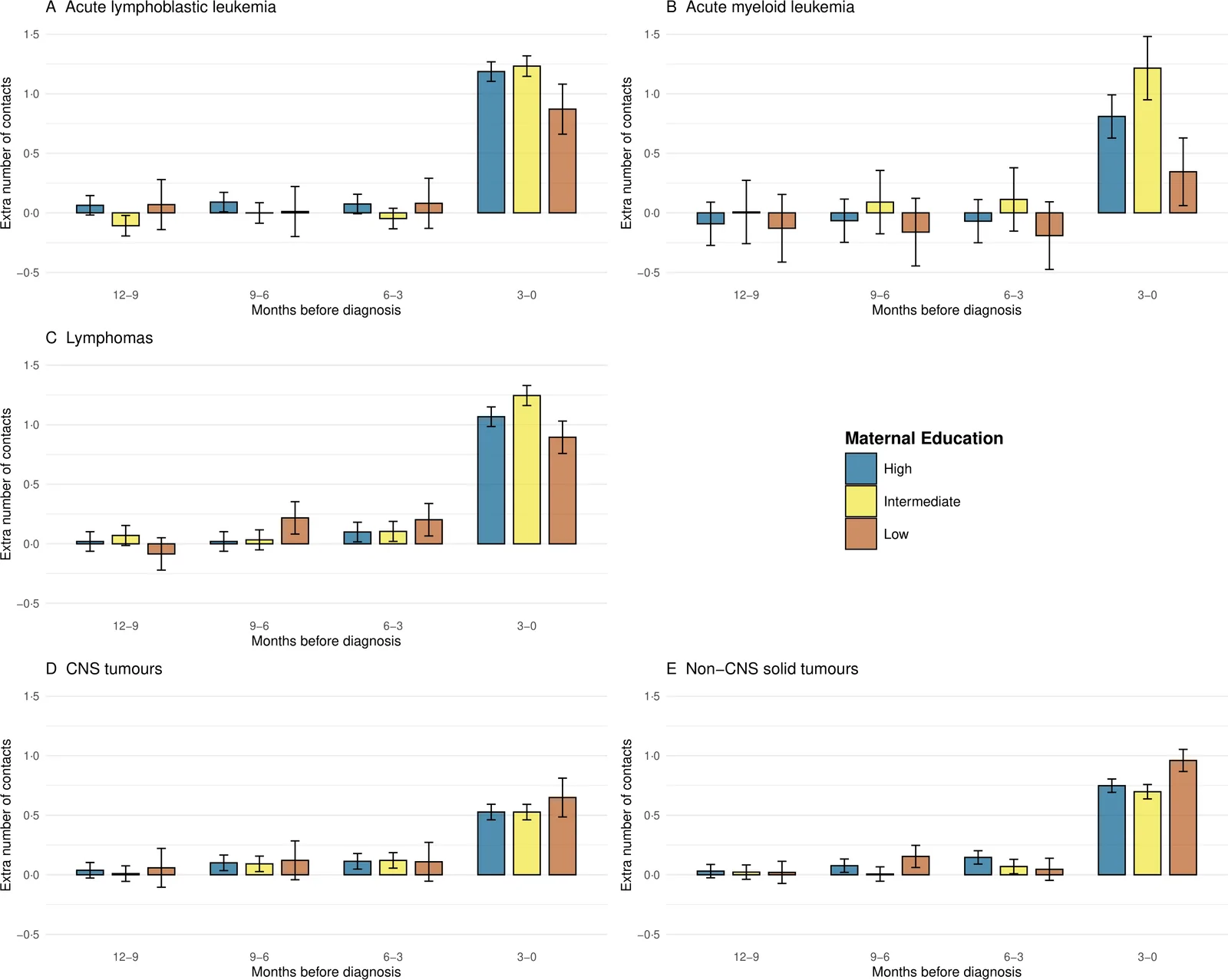
Additional primary healthcare contacts for childhood cancer patients before diagnosis across levels of maternal education, by 3-month intervals, stratified by diagnostic group. (A) Acute lymphoblastic leukaemia, (B) acute myeloid leukaemia, (C) lymphomas, (D) central nervous system (CNS) tumours and (E) non-CNS solid tumours.

Patterns by socioeconomic factors also varied. For haematological malignancies (ALL, AML and lymphomas), children whose mothers had a low educational background had the fewest additional PHC contacts, followed by those with highly educated mothers, while children of mothers with an intermediate educational background had the highest number, showing an inverted U-shaped pattern. In contrast, for solid tumours (both CNS and non-CNS), children of mothers with a low educational background had the highest number of additional PHC contacts during the last 3 months before diagnosis. This pattern was, however, not observed when maternal income was used instead of maternal education (online supplemental figure 2).

When focusing only on individuals with at least one PHC contact within the 3 months preceding the index date, we observed a small but statistically significant gradient in healthcare use among the population references, with higher utilisation among children of mothers with low educational backgrounds (figure 3). This pattern was not seen among childhood cancer patients, except for AML and CNS tumours. For AML, an inverted U-shaped pattern was again observed, with children of mothers with intermediate educational background having the highest number of contacts before diagnosis (aIRR_intermediate_=1.43; 95% CI 1.01 to 2.04), compared with children of mothers with a high socioeconomic background. In contrast, for CNS tumours, childhood cancer patients from families with low maternal education had a significantly higher number of contacts in the 3 months preceding diagnosis (aIRR_low_=1.28; 95% CI 1.00 to 1.62). Similar patterns were observed for the six-, nine- and twelve-month periods, although the effects were attenuated, reflecting the inclusion of other non-cancer related PHC contacts (online supplemental figures 3–5).

**Figure 3:**
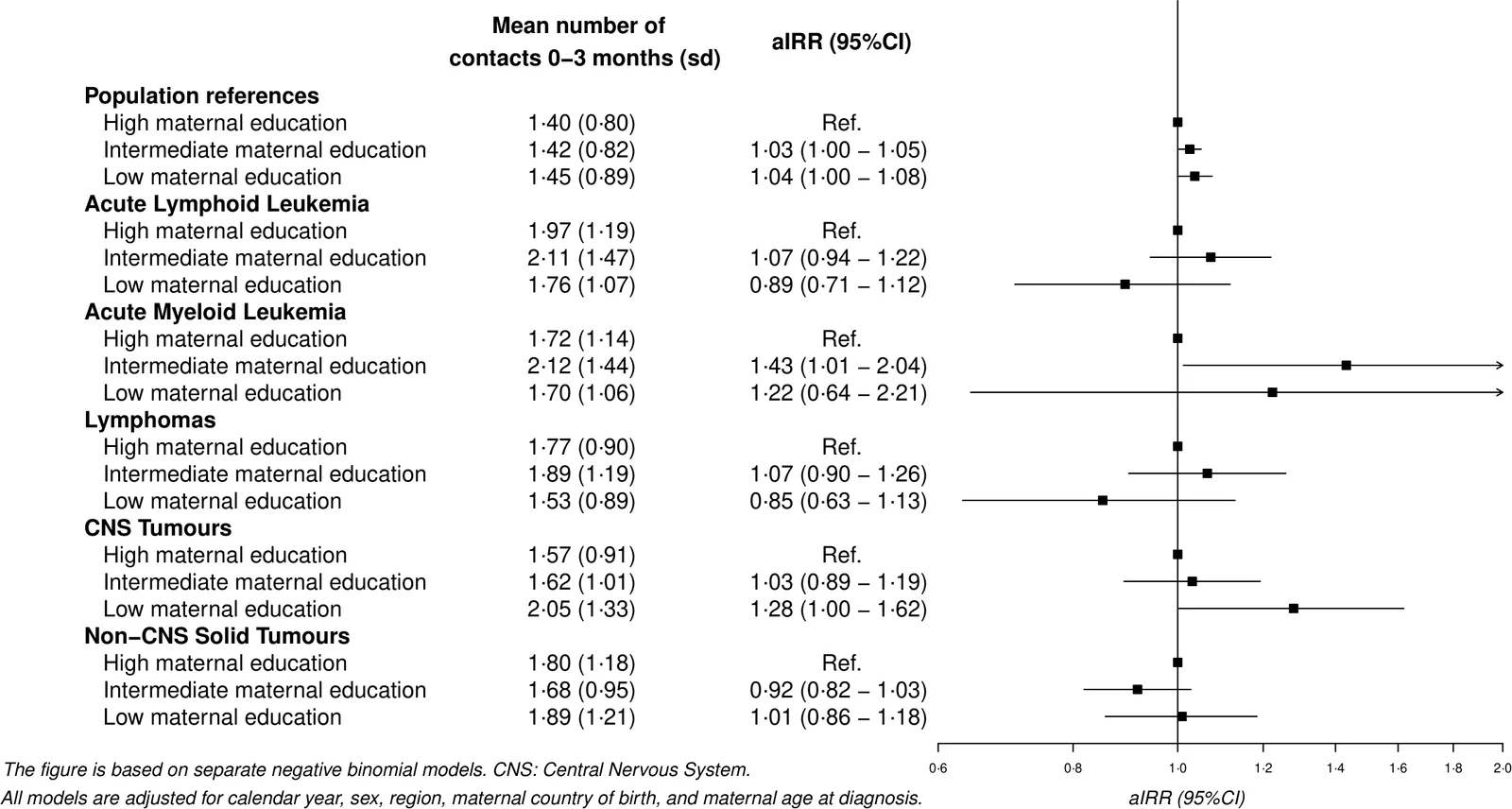
Adjusted incidence rate ratios (aIRR) for the number of contacts in the 3 months before diagnosis or index date across levels of maternal education, stratified by diagnostic group.

Regarding maternal income, a small but statistically significant inverse gradient in the frequency of PHC contacts was observed among population references. In contrast, among childhood cancer patients across all diagnostic groups, no statistically significant differences in contact frequency were observed across maternal income quintiles, with CIs consistently spanning the null value (online supplemental figure 6).

## Discussion

In this Swedish register-based study, we found that children with cancer had substantially more PHC contacts in the year before diagnosis than the general paediatric population, particularly during the 3 months before diagnosis, with marked variation across diagnostic groups. Notably, a considerable proportion of childhood cancer patients had no PHC visits prior to diagnosis. In the general paediatric population, clear differences in PHC utilisation were observed according to maternal educational level: children of mothers with lower educational attainment were more likely to have at least one PHC contact and, among PHC users, a higher frequency of PHC visits than those of highly educated mothers was observed. Among children with cancer, these educational gradients largely disappeared. The one exception was for CNS tumours, where PHC users from families with low maternal education had more PHC contacts 3 months before diagnosis.

Our findings are consistent with Pedersen *et al*,^12^ as they observed higher odds of frequent healthcare contacts during the 3 months preceding diagnosis among children with CNS tumours from lower educational backgrounds, with no strong SES gradients observed in other diagnostic groups. In contrast, our results differ from those of another Danish study, Friis Abrahamsen *et al*,^16^ who did not find socioeconomic differences in the odds of frequent PHC consultations among children with CNS tumours. Instead, they reported that childhood leukaemia patients whose parents had an intermediate educational level had the highest odds of frequent PHC consultations in the 3 months prior to diagnosis. Their study included all PHC modalities, including face-to-face, home, telephone and email consultations, and was not restricted to physician encounters only. This broader definition may explain their findings, as parents might seek non-physician or remote care for mild, non-specific leukaemia symptoms, consultation types where socioeconomic differences in utilisation are already well-documented.^26^

We found a high share of childhood cancer patients without any PHC contact 3 months before diagnosis, ranging from 22% in children with lymphomas to almost 50% in children with CNS tumours. This is consistent with one-county Swedish evidence from Thulesius *et al*^6^ who reported that one-third of children began their diagnostic process in specialist care. For CNS tumours, this high rate of direct presentation to hospital may be explained by children presenting with acute or severe symptoms (eg, signs of increased intracranial pressure) that prompt emergency care and therefore bypass PHC.

As expected, we found differences in PHC consultation rates by maternal education among the general Swedish paediatric population.^27 28^ Interestingly, we did not observe such educational gradients among childhood cancer patients. This suggests that the severity or nature of symptoms preceding a cancer diagnosis may override usual patterns of care-seeking driven by maternal education. Consequently, it appears that, at least in the Swedish context, primary care utilisation is not the primary driver of the survival disparities by maternal educational level reported for childhood cancers.

The exception of CNS tumours is notable. While many children bypass PHC due to acute symptoms, a significant subset follows a more prolonged diagnostic trajectory. This is frequently observed with low-grade gliomas, as typically slow-growing tumours, they often do not present with emergency symptoms. Instead, they manifest with vague, intermittent and non-specific signs, such as headache, nausea or behavioural changes, that are common in childhood and easily attributed to psychosocial or environmental causes. Our finding that children of mothers with low educational attainment have more PHC contacts 3 months before the diagnosis of a CNS tumour suggests that this diagnostic ambiguity may lead clinicians to interpret complaints in these families as benign or socially mediated, thereby increasing the number of consultations before a referral is made. In contrast, most leukaemia typically presents with objective or rapidly progressive signs, such as abnormal blood counts or palpable masses, which prompt timely diagnostic evaluation largely independent of family background. Consequently, the higher frequency of PHC consultations among children of mothers with lower education suggests that social factors may inadvertently prolong the diagnostic process for CNS tumours. However, further investigation is needed to replicate these findings and exclude chance associations.

This novel study is the first study to examine socioeconomic differences in PHC contacts among Swedish children with cancer, which may contribute to diagnostic delays. Our study is strengthened by the use of the National Cancer Register to identify cases, enabling a large sample size while minimising the risk of selection bias. However, some limitations should be noted. First, we could not determine whether PHC visits among childhood cancer patients were related to the cancer itself or to other health conditions. Nevertheless, the use of a population-based reference group allowed us to contextualise the patterns observed in children with cancer, although descriptively. Second, we could include information from only three regions, as these were the only ones with an available PHC registry. Although these regions cover 55% of the Swedish child population, they may underrepresent more rural areas of Sweden and therefore may not be fully representative of the national paediatric population. Third, this study focused exclusively on PHC contacts; contacts with secondary specialist care, tertiary services and paediatric emergency departments were not included. Because many acute paediatric oncology presentations involve direct or hospital-based routes to diagnosis, our findings are limited to the primary care pathway and cannot account for alternative pathways that may influence diagnostic intervals and survival rates. The use of registry data also limited the number of potential confounders we could adjust for; therefore, we cannot rule out residual confounding from factors such as parental mental or physical health or previous experiences with the healthcare system. Finally, the absence of socioeconomic differences in consultation rates does not necessarily indicate the absence of disparities in diagnostic delays, as this study did not analyse information on the interval from symptom onset or first symptom-related PHC visit to diagnosis.

Our findings highlight socioeconomic disparities in PHC utilisation that warrant clinical and policy attention. The observation that children with CNS tumours from lower socioeconomic backgrounds had more frequent PHC contacts prior to diagnosis suggests that diagnostic ambiguity may lead clinicians to misattribute early symptoms to benign or social factors, potentially lengthening the diagnostic pathway for these families. Conversely, the significant proportion of patients with no prior PHC contact raises questions about equitable access; it remains to be determined whether these children presented with more acute symptoms or if barriers such as the Swedish triage system, lack of trust or restricted personal networks influenced care-seeking. Identifying the mechanisms behind these divergent pathways, whether through protracted consultations or complete lack of primary care engagement, is crucial for developing targeted interventions that ensure the timeliness of a cancer diagnosis is not contingent on a family’s SES.

This register-based study demonstrates that, while disparities in PHC utilisation by maternal education are prevalent in the general Swedish paediatric population, these educational gradients largely disappear during the 12-month period prior to childhood cancer diagnosis, with the notable exception of CNS tumours. For children with CNS tumours, lower maternal education was associated with a higher frequency of PHC consultations, potentially signalling a more fragmented or delayed diagnostic pathway. While a lower frequency of prediagnostic PHC contacts was observed among families with higher maternal education, whether this reflects a true lower reliance on health services or an alternative care-seeking pathway—such as direct contact with secondary specialist or emergency care—remains an unmeasured hypothesis. Future research integrating secondary and tertiary care registers is needed to explore these diagnostic pathways further and to inform strategies aimed at promoting equitable and timely cancer diagnosis in children.

## Supplementary material

10.1136/bmjpo-2026-005059 online supplemental figure 1

## Data Availability

Data are available upon reasonable request.
