# Supplementary material for: Socioeconomic differences in primary healthcare use before childhood cancer diagnosis: a register-based cohort study in Sweden
Source: BMJ Paediatr Open. 2026 Sep 24;10(1):e005059. doi: 10.1136/bmjpo-2026-005059 (PMC13630025; doi:10.1136/bmjpo-2026-005059)
Supplement: Supplementary file 1 [file bmjpo-10-1-s001.pdf]

Supplementary figure 1. Adjusted odds ratios (aOR) of no PHC contacts in the 3 months before diagnosis or index date across levels of maternal income, stratified by diagnostic group.

	At least one contact 0−3 months / total No. (%)	aOR (95%CI)
**Population references**		
Income Q1 − Highest	4,595 / 21,497 (21·4%)	Ref.
Income Q2	4,003 / 17,916 (22·3%)	1·11 (1·06 − 1·17)
Income Q3	3,871 / 16,423 (23·6%)	1·14 (1·09 − 1·20)
Income Q4	4,474 / 16,763 (26·7%)	1·23 (1·17 − 1·29)
Income Q5−Lowest	5,214 / 18,589 (28·0%)	1·17 (1·11 − 1·23)
**Acute Lymphoid Leukemia**		
Income Q1 − Highest	120 / 149 (80·5%)	Ref.
Income Q2	90 / 119 (75·6%)	0·78 (0·43 − 1·41)
Income Q3	90 / 116 (77·6%)	0·84 (0·46 − 1·55)
Income Q4	128 / 162 (79·0%)	0·92 (0·51 − 1·63)
Income Q5−Lowest	131 / 178 (73·6%)	0·75 (0·43 − 1·31)
**Acute Myeloid Leukemia**		
Income Q1 − Highest	18 / 25 (72·0%)	Ref.
Income Q2	12 / 20 (60·0%)	0·57 (0·14 − 2·26)
Income Q3	19 / 22 (86·4%)	3·15 (0·63 − 19·25)
Income Q4	16 / 29 (55·2%)	0·55 (0·15 − 1·95)
Income Q5−Lowest	22 / 33 (66·7%)	1·05 (0·26 − 4·14)
**Lymphomas**		
Income Q1 − Highest	87 / 116 (75·0%)	Ref.
Income Q2	79 / 106 (74·5%)	0·93 (0·50 − 1·73)
Income Q3	64 / 81 (79·0%)	1·20 (0·59 − 2·48)
Income Q4	53 / 70 (75·7%)	0·98 (0·48 − 2·05)
Income Q5−Lowest	62 / 74 (83·8%)	1·61 (0·74 − 3·65)
**CNS Tumours**		
Income Q1 − Highest	127 / 278 (45·7%)	Ref.
Income Q2	121 / 213 (56·8%)	1·54 (1·07 − 2·23)
Income Q3	90 / 163 (55·2%)	1·39 (0·94 − 2·08)
Income Q4	92 / 174 (52·9%)	1·23 (0·83 − 1·82)
Income Q5−Lowest	99 / 180 (55·0%)	1·25 (0·83 − 1·88)
**Non−CNS Solid Tumours**		
Income Q1 − Highest	209 / 352 (59·4%)	Ref.
Income Q2	178 / 266 (66·9%)	1·38 (0·99 − 1·93)
Income Q3	137 / 224 (61·2%)	1·04 (0·73 − 1·48)
Income Q4	156 / 236 (66·1%)	1·23 (0·86 − 1·77)
Income Q5−Lowest	170 / 262 (64·9%)	1·08 (0·75 − 1·56)

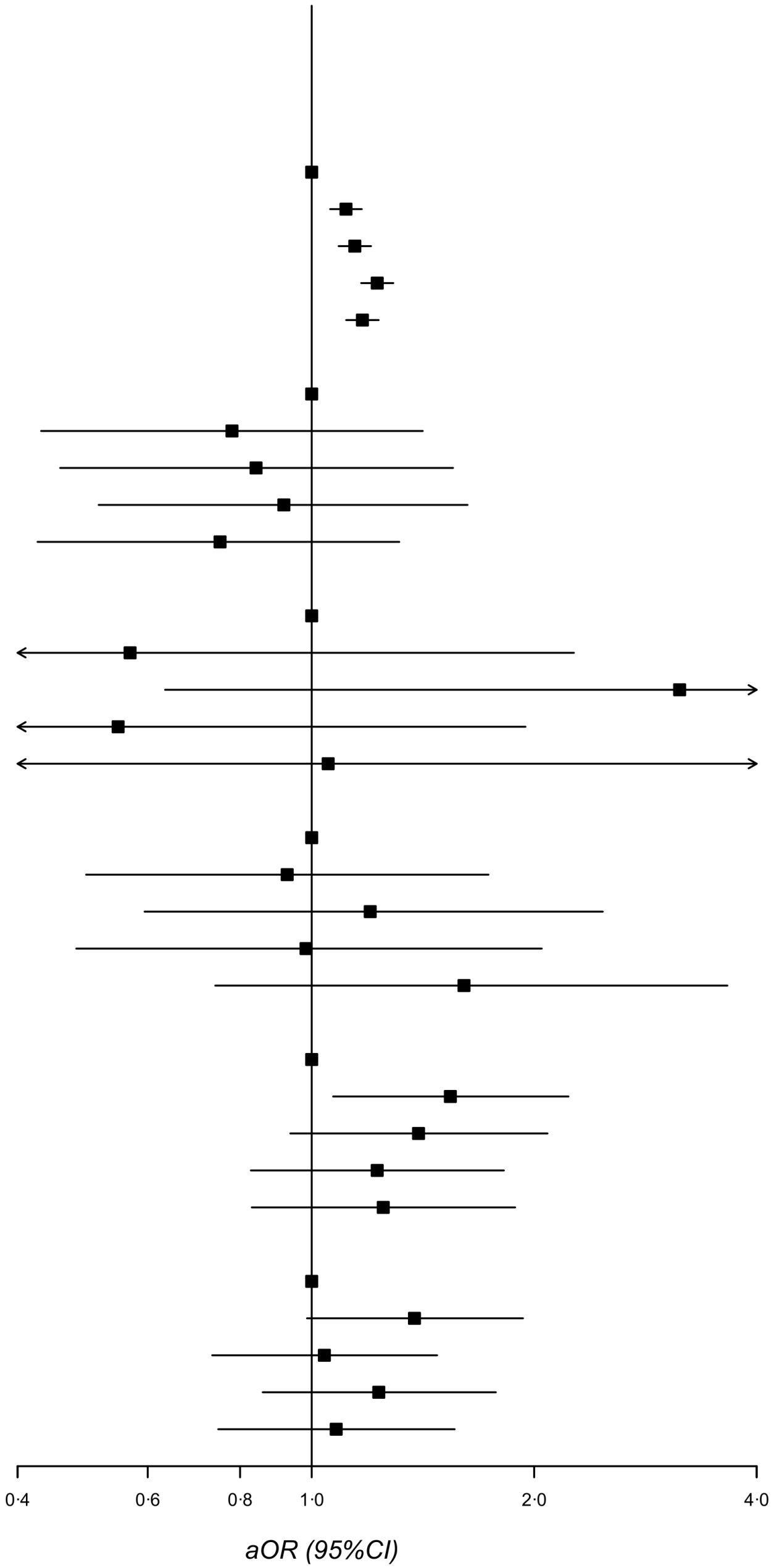

The figure is based on separate logistic regression models. CNS: Central Nervous System.

All models are adjusted for calendar year, sex, region, maternal country of birth, and maternal age at diagnosis.

Supplementary figure 2. Additional primary health care contacts for childhood cancer patients before diagnosis across levels of maternal income, by three-month intervals, stratified by diagnostic group.

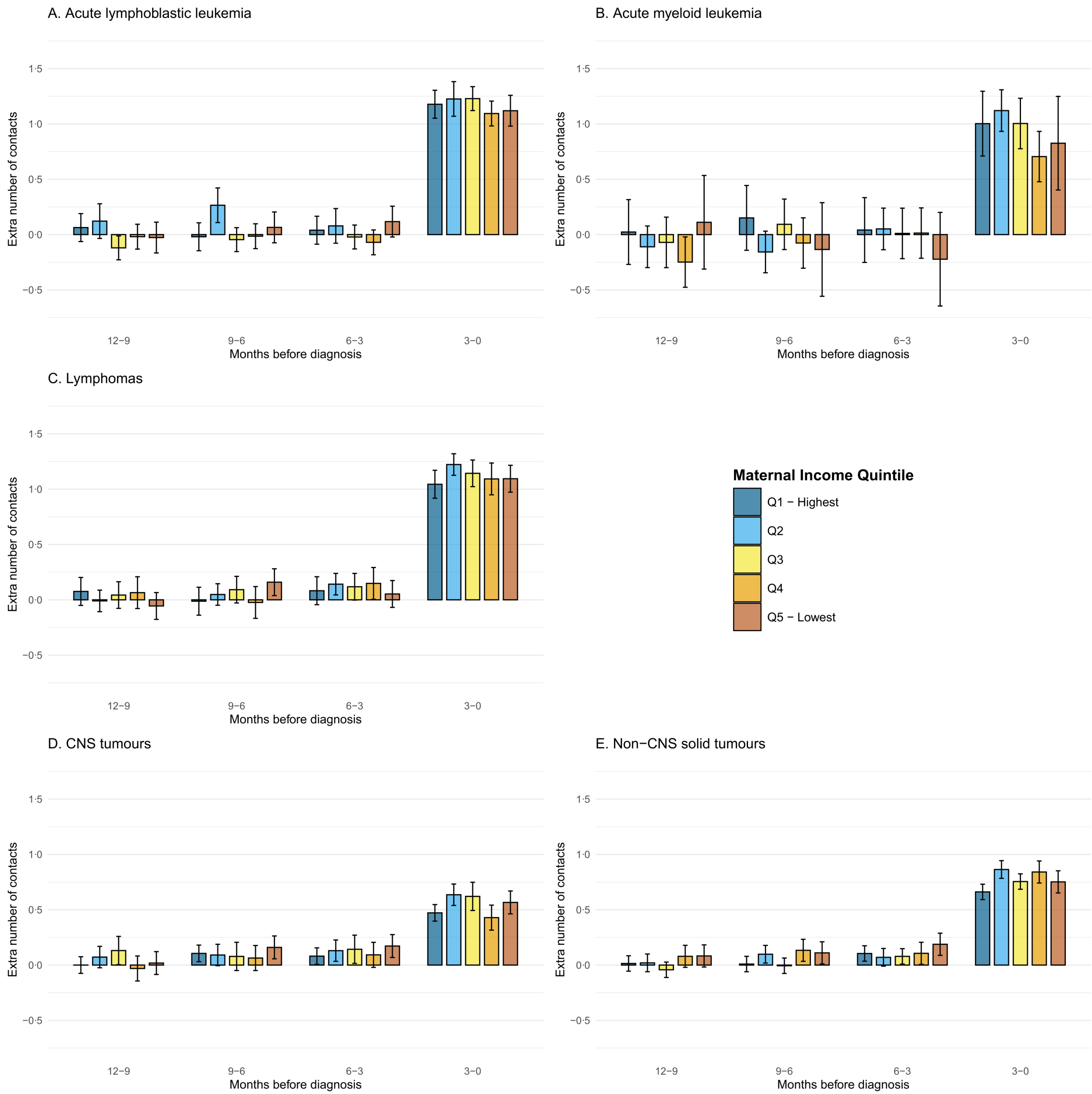

Supplementary figure 3. Adjusted incidence rate ratios (aIRR) for the number of contacts in the 6 months before diagnosis or index date across levels of maternal education, stratified by diagnostic group.

	Mean number of contacts 0−6 months (sd)	aIRR (95%CI)
**Population references**		
High maternal education	1·70 (1·15)	Ref.
Intermediate maternal education	1·72 (1·18)	1·03 (1·01 − 1·05)
Low maternal education	1·79 (1·30)	1·05 (1·02 − 1·08)
**Acute Lymphoid Leukemia**		
High maternal education	2·38 (1·45)	Ref.
Intermediate maternal education	2·43 (1·79)	1·03 (0·92 − 1·16)
Low maternal education	2·19 (1·52)	0·91 (0·74 − 1·10)
**Acute Myeloid Leukemia**		
High maternal education	1·90 (1·26)	Ref.
Intermediate maternal education	2·45 (2·02)	1·42 (1·04 − 1·95)
Low maternal education	1·82 (1·33)	1·08 (0·59 − 1·88)
**Lymphomas**		
High maternal education	2·09 (1·20)	Ref.
Intermediate maternal education	2·18 (1·44)	1·05 (0·90 − 1·22)
Low maternal education	1·82 (1·19)	0·86 (0·66 − 1·10)
**CNS Tumours**		
High maternal education	1·88 (1·26)	Ref.
Intermediate maternal education	1·98 (1·34)	1·06 (0·94 − 1·20)
Low maternal education	2·25 (1·35)	1·17 (0·95 − 1·42)
**Non−CNS Solid Tumours**		
High maternal education	2·20 (1·62)	Ref.
Intermediate maternal education	1·95 (1·23)	0·88 (0·80 − 0·97)
Low maternal education	2·25 (1·56)	0·98 (0·85 − 1·13)

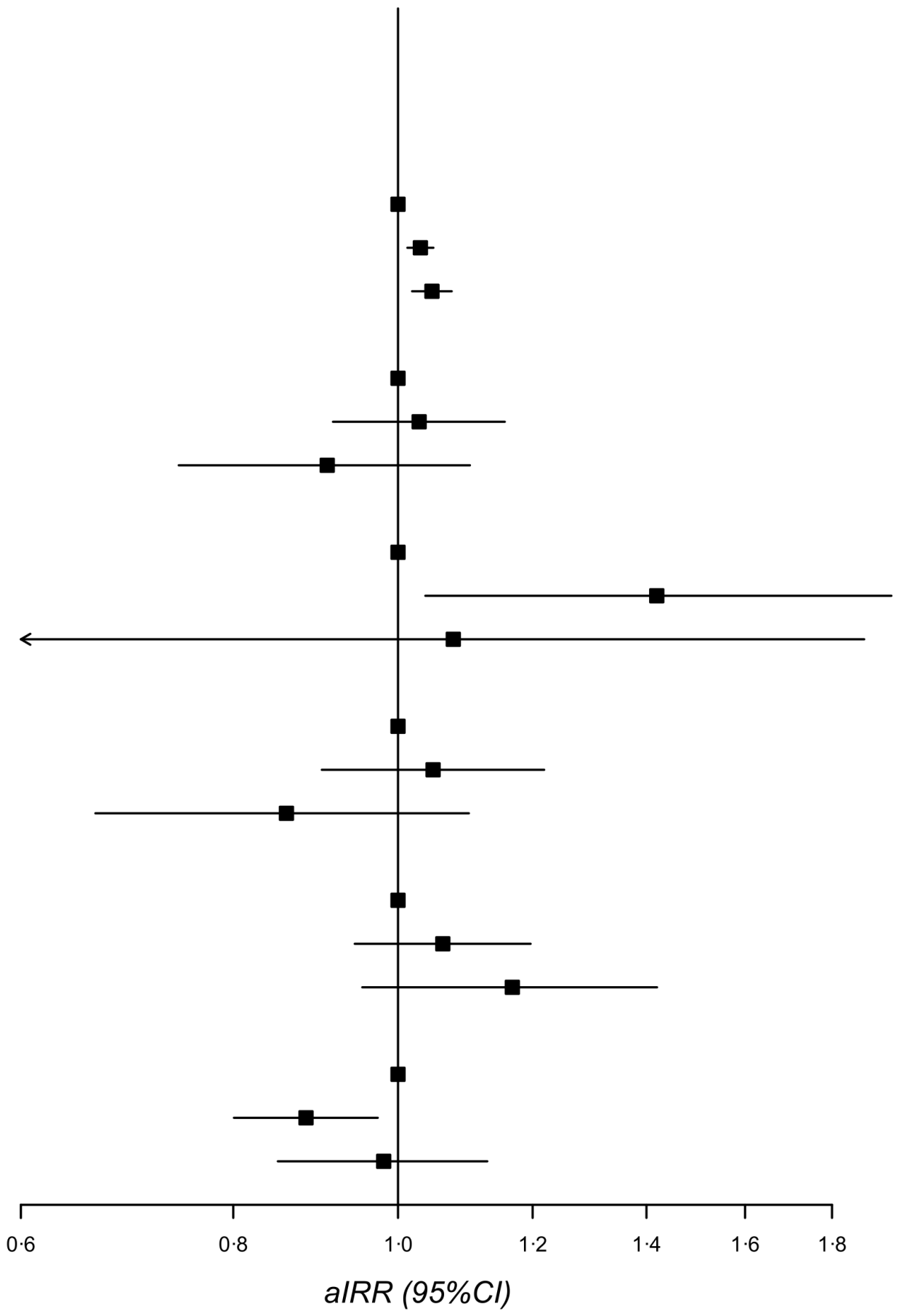

The figure is based on separate negative binomial models. CNS: Central Nervous System.
All models are adjusted for calendar year, sex, region, maternal country of birth, and maternal age at diagnosis.

Supplementary figure 4. Adjusted incidence rate ratios (aIRR) for the number of contacts in the 9 months before diagnosis or index date across levels of maternal education, stratified by diagnostic group.

	Mean number of contacts 0−9 months (sd)	aIRR (95%CI)
**Population references**		
High maternal education	1·99 (1·43)	Ref.
Intermediate maternal education	2·03 (1·50)	1·05 (1·03 − 1·06)
Low maternal education	2·09 (1·65)	1·06 (1·03 − 1·08)
**Acute Lymphoid Leukemia**		
High maternal education	2·76 (1·74)	Ref.
Intermediate maternal education	2·77 (2·10)	1·02 (0·91 − 1·14)
Low maternal education	2·59 (1·69)	0·93 (0·77 − 1·12)
**Acute Myeloid Leukemia**		
High maternal education	2·22 (1·51)	Ref.
Intermediate maternal education	2·98 (2·09)	1·50 (1·13 − 1·99)
Low maternal education	2·27 (1·62)	1·14 (0·66 − 1·89)
**Lymphomas**		
High maternal education	2·34 (1·31)	Ref.
Intermediate maternal education	2·46 (1·60)	1·05 (0·91 − 1·21)
Low maternal education	2·32 (1·52)	0·96 (0·76 − 1·21)
**CNS Tumours**		
High maternal education	2·20 (1·66)	Ref.
Intermediate maternal education	2·32 (1·75)	1·07 (0·96 − 1·19)
Low maternal education	2·48 (1·40)	1·10 (0·91 − 1·32)
**Non−CNS Solid Tumours**		
High maternal education	2·52 (1·84)	Ref.
Intermediate maternal education	2·26 (1·61)	0·91 (0·83 − 1·00)
Low maternal education	2·66 (2·01)	1·04 (0·91 − 1·20)

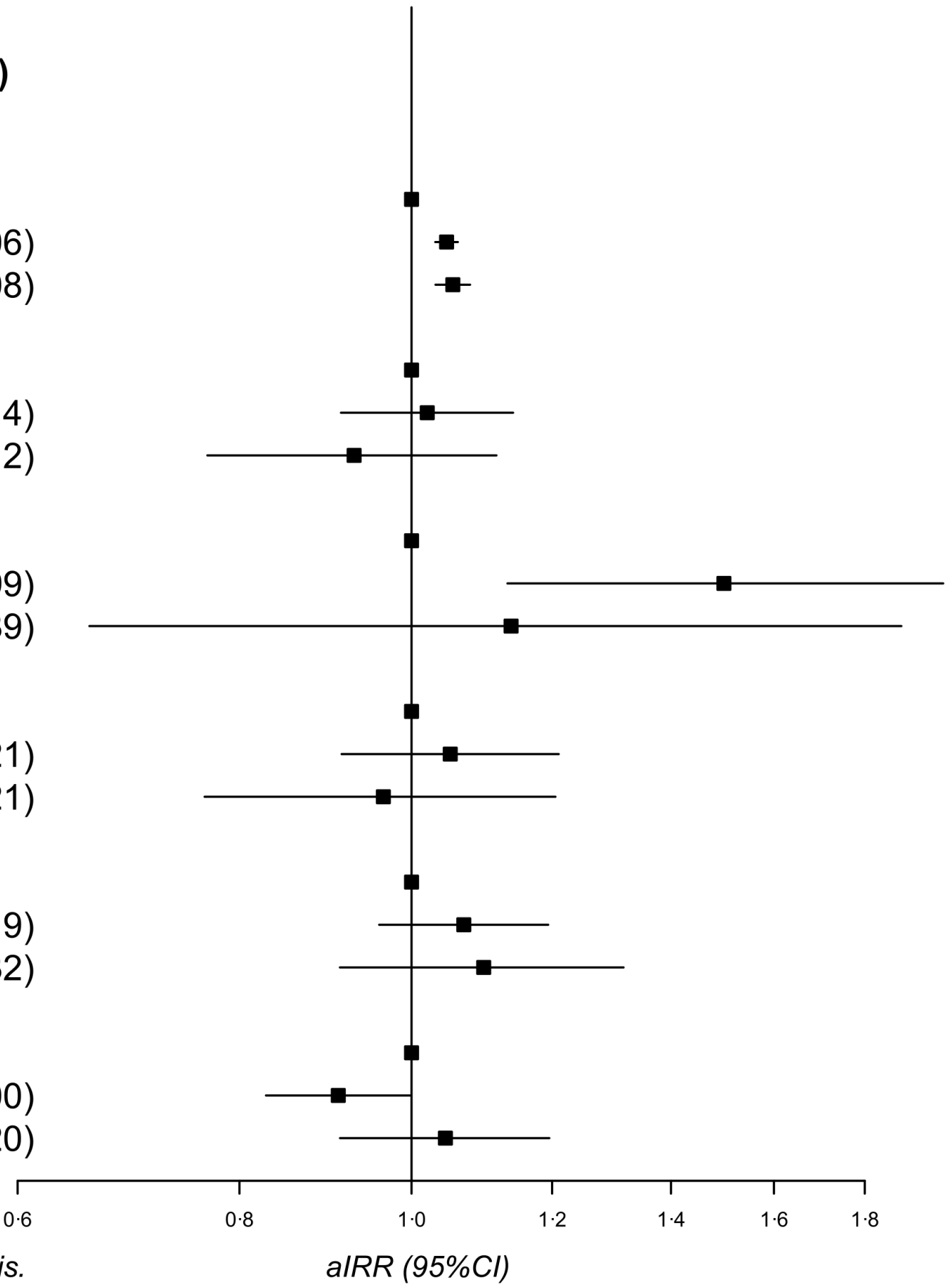

The figure is based on separate negative binomial models. CNS: Central Nervous System.

All models are adjusted for calendar year, sex, region, maternal country of birth, and maternal age at diagnosis.

Supplementary figure 5. Adjusted incidence rate ratios (aIRR) for the number of contacts in the 12 months before diagnosis or index date across levels of maternal education, stratified by diagnostic group.

	Mean number of contacts 0−12 months (sd)	aIRR (95%CI)
**Population references**		
High maternal education	2·90 (2·06)	Ref.
Intermediate maternal education	3·00 (2·18)	1·06 (1·04 − 1·08)
Low maternal education	3·12 (2·44)	1·08 (1·05 − 1·11)
**Acute Lymphoid Leukemia**		
High maternal education	3·31 (2·06)	Ref.
Intermediate maternal education	3·21 (2·48)	0·98 (0·87 − 1·10)
Low maternal education	3·29 (2·24)	0·97 (0·80 − 1·17)
**Acute Myeloid Leukemia**		
High maternal education	2·67 (1·80)	Ref.
Intermediate maternal education	3·50 (2·48)	1·49 (1·12 − 2·00)
Low maternal education	2·30 (1·89)	1·05 (0·57 − 1·81)
**Lymphomas**		
High maternal education	2·64 (1·50)	Ref.
Intermediate maternal education	2·86 (1·76)	1·07 (0·93 − 1·22)
Low maternal education	2·55 (1·64)	0·95 (0·75 − 1·19)
**CNS Tumours**		
High maternal education	2·83 (2·13)	Ref.
Intermediate maternal education	2·90 (2·21)	1·02 (0·90 − 1·16)
Low maternal education	3·07 (1·63)	1·05 (0·84 − 1·30)
**Non−CNS Solid Tumours**		
High maternal education	3·08 (2·17)	Ref.
Intermediate maternal education	2·80 (1·99)	0·92 (0·84 − 1·02)
Low maternal education	3·25 (2·23)	1·03 (0·89 − 1·18)

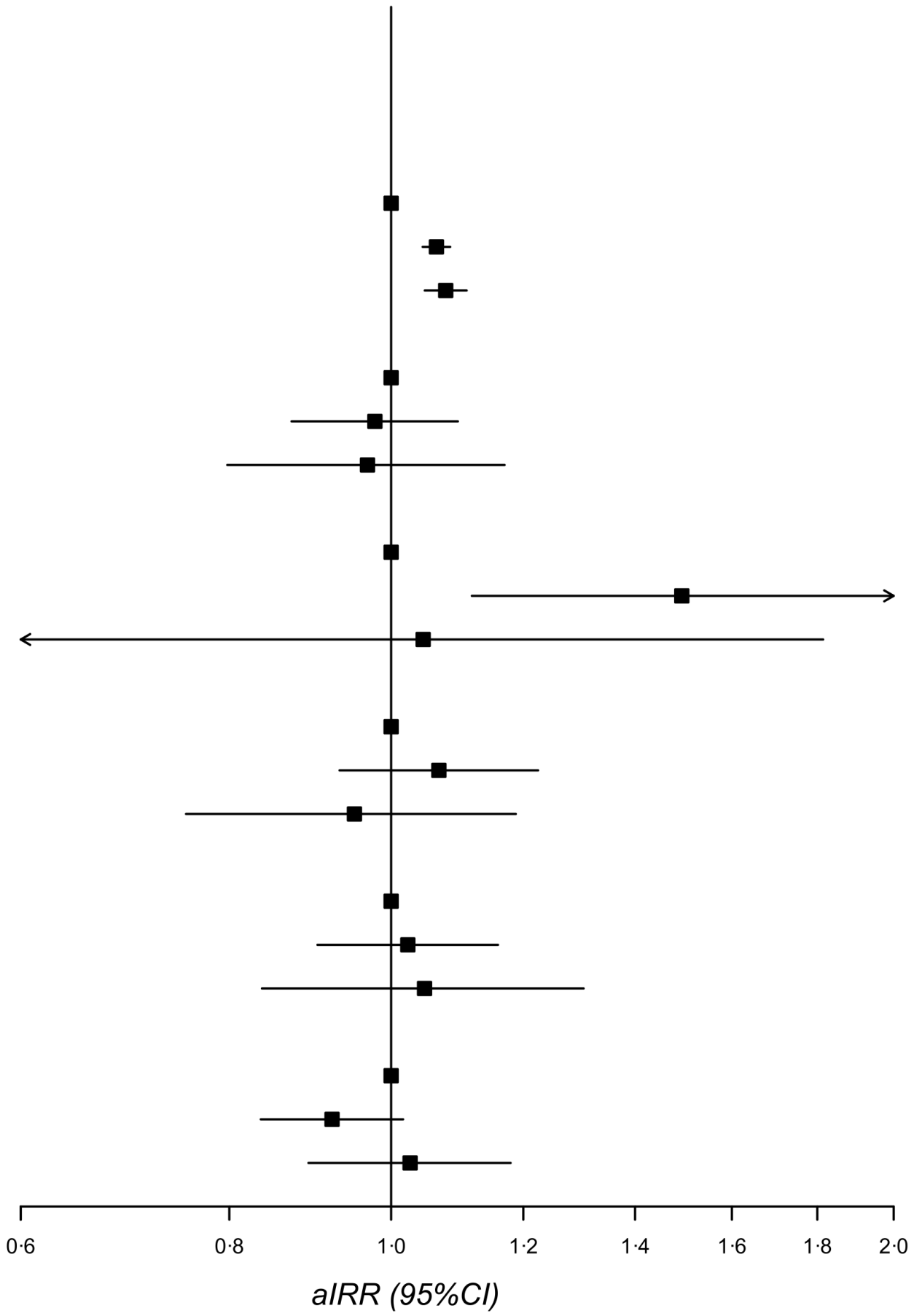

The figure is based on separate negative binomial models. CNS: Central Nervous System.
All models are adjusted for calendar year, sex, region, maternal country of birth, and maternal age at diagnosis.

Supplementary figure 6. Adjusted odds ratios (aOR) of no PHC contacts in the 3 months before diagnosis or index date across levels of maternal income, stratified by diagnostic group.

	Mean number of contacts 0−3 months (sd)	aIRR (95%CI)
**Population references**		
Income Q1 − Highest	1·36 (0·72)	Ref.
Income Q2	1·38 (0·78)	1·03 (0·99 − 1·07)
Income Q3	1·42 (0·85)	1·05 (1·01 − 1·09)
Income Q4	1·45 (0·85)	1·06 (1·02 − 1·10)
Income Q5−Lowest	1·47 (0·90)	1·05 (1·01 − 1·08)
**Acute Lymphoid Leukemia**		
Income Q1 − Highest	1·85 (1·10)	Ref.
Income Q2	2·08 (1·31)	1·14 (0·93 − 1·38)
Income Q3	2·09 (1·50)	1·16 (0·96 − 1·42)
Income Q4	1·91 (1·11)	1·02 (0·85 − 1·23)
Income Q5−Lowest	2·10 (1·44)	1·12 (0·93 − 1·36)
**Acute Myeloid Leukemia**		
Income Q1 − Highest	1·89 (0·96)	Ref.
Income Q2	2·50 (2·20)	1·37 (0·81 − 2·30)
Income Q3	1·58 (1·30)	0·96 (0·57 − 1·63)
Income Q4	2·00 (1·03)	1·14 (0·67 − 1·92)
Income Q5−Lowest	1·95 (1·05)	0·92 (0·55 − 1·54)
**Lymphomas**		
Income Q1 − Highest	1·74 (0·95)	Ref.
Income Q2	2·00 (1·23)	1·16 (0·92 − 1·45)
Income Q3	1·77 (0·99)	1·04 (0·81 − 1·34)
Income Q4	1·79 (0·99)	1·04 (0·80 − 1·36)
Income Q5−Lowest	1·66 (0·96)	0·98 (0·75 − 1·27)
**CNS Tumours**		
Income Q1 − Highest	1·62 (1·06)	Ref.
Income Q2	1·63 (0·92)	0·97 (0·80 − 1·19)
Income Q3	1·68 (1·04)	1·00 (0·81 − 1·24)
Income Q4	1·48 (0·80)	0·86 (0·68 − 1·07)
Income Q5−Lowest	1·72 (1·10)	0·97 (0·78 − 1·20)
**Non−CNS Solid Tumours**		
Income Q1 − Highest	1·62 (0·95)	Ref.
Income Q2	1·76 (1·06)	1·08 (0·93 − 1·27)
Income Q3	1·81 (1·17)	1·10 (0·93 − 1·30)
Income Q4	1·92 (1·26)	1·16 (0·98 − 1·36)
Income Q5−Lowest	1·85 (1·13)	1·07 (0·91 − 1·27)

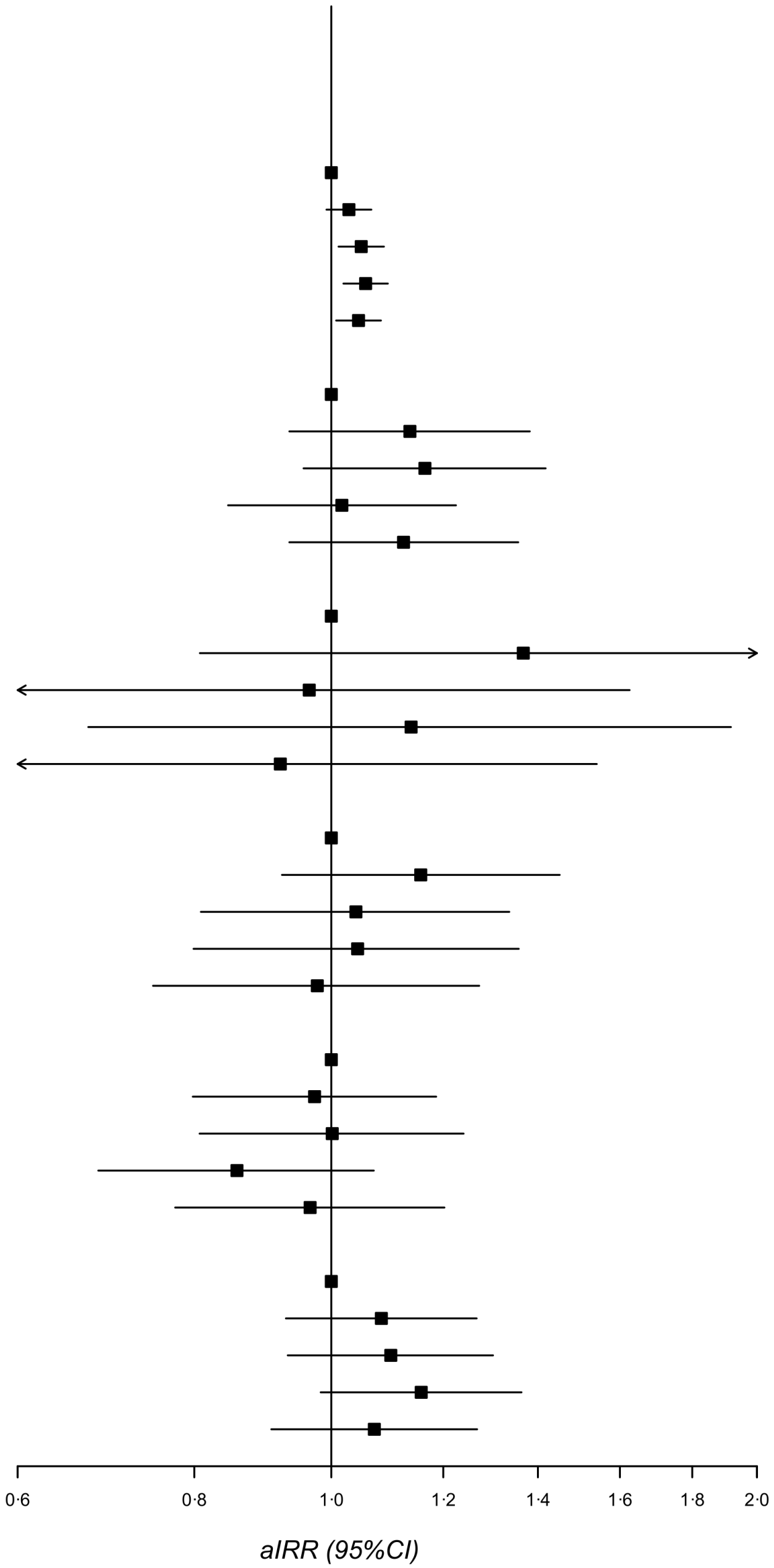

The figure is based on separate logistic regression models. CNS: Central Nervous System.

All models are adjusted for calendar year, sex, region, maternal country of birth, and maternal age at diagnosis.
