# Supplementary material for: Socioeconomic differences in primary healthcare use before childhood cancer diagnosis: a register-based cohort study in Sweden
Source: BMJ Paediatr Open. 2026 Sep 24;10(1):e005059. doi: 10.1136/bmjpo-2026-005059 (PMC13630025; doi:10.1136/bmjpo-2026-005059)
Supplement: Supplementary file 2 [file bmjpo-2026-005059.reviewer_comments.pdf]

PEER REVIEW HISTORY

BMJ Paediatrics Open publishes all reviews undertaken for accepted manuscripts. Reviewers are asked to complete a checklist review form and are provided with free text boxes to elaborate on their assessment. These free text comments are reproduced below.

ARTICLE DETAILS

**TITLE (PROVISIONAL)**	Socioeconomic differences in primary health care use before childhood cancer diagnosis: A register-based cohort study in Sweden
**AUTHORS**	Reißinger, Julia<br>Kampitsi, Christina-Evmorfia<br>Feychting, Maria<br>Mogensen, Hanna<br>Rossides, Marios<br>Chen, Eric<br>Harila, Arja H.<br>Georgantzi, Kleopatra<br>Tettamanti, Giorgio<br>Louro, Javier

VERSION 1 – REVIEW

**REVIEWER NAME**	Harron, Katie
**REVIEWER AFFILIATION**	UCL Great Ormond Street Institute of Child Health
**REVIEWER CONFLICT OF INTEREST**	No Conflict of Interest
**AI DISCLOSURE**	No, I have not used AI
**DATE REVIEW RETURNED**	21-Jul-2026

**GENERAL COMMENTS**	Abstract: You provide the absolute results for the mean contacts for each group, but can you say whether there was any evidence of a difference between these groups, i.e. by providing the mean difference and confidence interval for the difference (see e.g. section on subgroup analysis here https://doi.org/10.1136/bmj-2022-072883).<br><br>If the aim is to determine whether the effect of deprivation on PHC is consistent between cases and controls (which is how the results are presented) then rather than stratifying the analyses and treating the groups as separate, you need to use an interaction term to test whether there were significant differences in patterns across deprivation groups between cases/controls.<br><br>For the secondary analysis looking at frequency of contacts, I understand that children with no contacts were removed from this analysis. Presumably this applies to both controls and cases. How did this affect the matching / balance between groups? If there were differences in the proportion with 1 or more contacts between groups (and this should be reported, which I don't think it currently is within the results, nor the numbers in the sub-analysis), the matched groups may no longer be balanced and you would need to account for this, e.g. by creating a new matched cohort of just those who had at least one contact for this analysis.

	Was the frequency of PHC contacts normally distributed?<br><br>How were missing data treated within models?<br><br>I understand that maternal education was chosen a priori as the main exposure. However, the text tends to refer to "socioeconomic factors", for example "intermediate SES". Clear and consistent terminology should be used throughout to make clear when you are talking about maternal education.<br><br>There is in fact a clear pattern in number of PHC by maternal income, which mirrors the pattern seen in the non-cancer group. This is not really described in the main text. I would like to see this described further as I think it adds context to the education findings.<br><br>"This register-based study demonstrates that, while socioeconomic disparities in PHC utilization are prevalent in the general Swedish paediatric population, these gradients largely disappear following the onset of childhood cancer symptoms" I don't think you can conclude this, as you don't have information about when cancer symptoms began. All you can describe is what happened in the 12 months prior to diagnosis.<br><br>"families from higher socioeconomic backgrounds may be more likely to bypass primary care in favour of direct specialist or emergency contact." Again I don't think you can infer this from the data that were analysed as you don't have any information on specialist care.<br><br>Figure 1 and Figure 3: x axis needs to be labelled.

VERSION 1 – AUTHOR RESPONSE

Reviewer 1

Comments to the Author

1. Abstract: You provide the absolute results for the mean contacts for each group, but can you say whether there was any evidence of a difference between these groups, i.e. by providing the mean difference and confidence interval for the difference (see e.g. section on subgroup analysis here https://doi.org/10.1136/bmj-2022-072883).

Response: We thank the reviewer for this helpful suggestion to report the effect size and uncertainty for group comparators. In accordance with this recommendation, we have updated the Abstract to present the mean difference, its 95% confidence interval, and the corresponding p-value comparing prediagnostic primary healthcare contacts between children with cancer and population references.

New text (Abstract – clean version line 51): Children with cancer had more PHC contacts than population references in the year before diagnosis (mean 2·28 vs 1·27, mean difference 1·01, 95% confidence interval to 0·94 to 1·08; P<0·001)

2. If the aim is to determine whether the effect of deprivation on PHC is consistent between cases and controls (which is how the results are presented) then rather than stratifying the analyses and treating

the groups as separate, you need to use an interaction term to test whether there were significant differences in patterns across deprivation groups between cases/controls.

Response: We thank the reviewer for this constructive insight regarding the evaluation of group differences. Our primary aim was to replicate and build upon prior findings from Denmark (Pedersen et al., 2021; Abrahamsen et al., 2018) by investigating whether socioeconomic position influences prediagnostic PHC utilization specifically among children with cancer in Sweden. The comparator group was included primarily to provide baseline contextual figures for the general population, rather than to establish a formal statistical contrast in the effect magnitude between groups. To avoid implying a formal statistical comparison of effect sizes between cases and population references without a dedicated interaction test, we have revised the manuscript text (specifically in the statistical analysis section) to clarify that results are presented within each group independently.

Pedersen LH, Erdmann F, Aalborg GL, Hjalgrim LL, Larsen HB, Schmiegelow K, et al. Socioeconomic position and prediagnostic health care contacts in children with cancer in Denmark: a nationwide register study. BMC Cancer. 2021;21(1):1104.

Friis Abrahamsen C, Ahrensberg JM, Vedsted P. Utilisation of primary care before a childhood cancer diagnosis: do socioeconomic factors matter?: A Danish nationwide population-based matched cohort study. BMJ Open. 2018;8(8):e023569.

New text (Methods – clean version line 232): Analyses were stratified by case-population references status to assess socioeconomic gradients within each cohort independently. Matched population references served to establish population-level baseline reference patterns rather than to conduct formal effect-modification testing. As interaction terms were not included in the main models, relative differences in effect magnitude between cases and controls are presented for contextual baseline comparison.

3. For the secondary analysis looking at frequency of contacts, I understand that children with no contacts were removed from this analysis. Presumably this applies to both controls and cases. How did this affect the matching / balance between groups? If there were differences in the proportion with 1 or more contacts between groups (and this should be reported, which I don't think it currently is within the results, nor the numbers in the sub-analysis), the matched groups may no longer be balanced and you would need to account for this, e.g. by creating a new matched cohort of just those who had at least one contact for this analysis.

Response: We sincerely thank the reviewer for this comment and for raising such an important point regarding our contact frequency analysis. We truly appreciate the opportunity to clarify our analytical framework and improve the transparency of our manuscript.

To address this query, we would first like to clarify that our study was a nationwide matched population-based cohort where childhood cancer status represents the cohort exposure group rather than an outcome in a traditional case-control design. The initial 1:25 matching on sex, region, and birth date was established at the cohort entry stage to select population references from the Total Population Register.

Regarding the negative binomial analysis, this is an integral part of our main analytical framework, which follows a standard two-part (hurdle) approach to model healthcare utilization. The logistic regression first models the probability of having any vs. no contacts, whereas the negative binomial model evaluates contact frequency strictly among active healthcare users. We deliberately restricted this

second step to individuals with at least one contact to ensure that the findings from the logistic regression were not duplicated or confounded in the count analysis.

We completely agree with the reviewer on the necessity of accounting for potential imbalances when restricting analyses to subgroups. To ensure robust control, all our negative binomial regression models are fully multivariable adjusted for all the original matching variables (sex, region, calendar year, and age at index date) as well as key socioeconomic and demographic factors. This multivariable adjustment appropriately controls any differences in baseline characteristics within the user subgroup without requiring a new matched sample, which would alter the underlying population cohort structure.

The reviewer's suggestion to report these exact subgroup numbers is extremely helpful. The exact absolute numbers (n) and proportions (%) of both cases (and subgroups) and population references who had at least one primary health care contact during the 3-month prediagnostic window—and who were subsequently included in this negative binomial analysis—are detailed across all categories in Figure 1. We have now updated the text in the Results section to explicitly guide the reader to Figure 1 for these specific subgroup counts and percentages.

New text (Results – clean version line 257): Absolute numbers and proportions of individuals with at least one PHC contact across categories are detailed in Figure 1.

4. Was the frequency of PHC contacts normally distributed?

Response: We thank the reviewer for this question. The frequency of primary health care (PHC) contacts was not normally distributed. As is typical for healthcare utilization and contact count data, the distribution was strongly right-skewed, characterized by a large proportion of individuals with zero contacts and a long right tail of individuals with multiple contacts, leading to significant overdispersion (where the variance exceeded the mean).

Because standard linear regression models relying on normality assumptions are inappropriate for such data, we selected negative binomial regression models to appropriately account for the non-normal, right-skewed, and overdispersed distribution of count data.

To ensure complete clarity for readers, we have updated the Methods section to explicitly describe the underlying distribution of the contact data and justify the model selection.

New text (Methods – clean version line 224): This model was selected to account for the non-normal, right-skewed distribution and overdispersion of the contact count data.

5. How were missing data treated within models?

Response: We thank the reviewer for this question regarding missing data.

Missing data in our study were minimal and restricted exclusively to the maternal socioeconomic status (SES) variables extracted from the longitudinal integrated database (LISA) register. Specifically, the proportion of missing values was approximately 3% for maternal education, 2% for maternal income, and <2% for maternal country of birth. There were no missing data for primary health care contacts, age, sex, or region.

Because the proportion of missingness across all variables was exceptionally low (<2–3%), a complete-case analysis was performed, as the potential bias introduced by missing values at this level is negligible and unlikely to affect the point estimates or statistical inferences.

To ensure full clarity for readers and reviewers, in table 1 the exact amount of missing data per variable is detailed and in the statistical analysis a statement about the complete case analysis is included.

6. I understand that maternal education was chosen a priori as the main exposure. However, the text tends to refer to "socioeconomic factors", for example "intermediate SES". Clear and consistent terminology should be used throughout to make clear when you are talking about maternal education.

Response: We thank the reviewer for this excellent observation regarding terminology. We completely agree that precise and consistent terminology is essential to avoid ambiguity, given that maternal education was our pre-specified primary indicator of socioeconomic position.

In response to this comment, **we have thoroughly revised the entire manuscrip**t to ensure that we consistently refer to **"**maternal education**"** (e.g., *"low maternal education"*, *"intermediate maternal education"*, or *"maternal educational background"*) whenever presenting or discussing findings based on education, reserving terms such as "maternal income" specifically for the secondary analyses. **We have removed ambiguous phrasing** such as "intermediate SES" or "socioeconomic factors" where it referred specifically to maternal education in many sections of the article.

We are very grateful for this suggestion, as it has substantially improved the clarity and precision of our paper.

7. There is in fact a clear pattern in number of PHC by maternal income, which mirrors the pattern seen in the non-cancer group. This is not really described in the main text. I would like to see this described further as I think it adds context to the education findings.

Response: We thank the reviewer for this careful reading of the secondary analysis on maternal income. Upon closer inspection of the numerical estimates and 95% confidence intervals (aIRRs) presented in Supplementary Figure 6, a small but statistically significant gradient is indeed present among the general population references. However, among childhood cancer patients, point estimates across maternal income quintiles fluctuate around unity, and all 95% confidence intervals cross 1.00 across all diagnostic subgroups, indicating no statistically significant association between maternal income and contact frequency in the cancer cohort.

To ensure complete clarity and prevent any misinterpretation of these findings, we have updated the text in the Results section to explicitly detail both the modest gradient observed in the general population references and the lack of statistically significant differences among childhood cancer patients.

New text (Results – clean version line 305): Regarding maternal income, a small but statistically significant inverse gradient in the frequency of PHC contacts was observed among population references. In contrast, among childhood cancer patients across all diagnostic groups, no statistically significant differences in contact frequency were observed across maternal income quintiles, with confidence intervals consistently spanning the null value (Supplementary Figure 6).

8. “This register-based study demonstrates that, while socioeconomic disparities in PHC utilization are prevalent in the general Swedish paediatric population, these gradients largely disappear following the onset of childhood cancer symptoms” I don’t think you can conclude this, as you don’t have information about when cancer symptoms began. All you can describe is what happened in the 12 months prior to diagnosis.

Response: We fully agree with the reviewer and thank them for pointing out this critical distinction. Because our data are drawn from administrative health registers, we do not have exact data on the precise onset of clinical symptoms for individual patients, but rather evaluate healthcare utilization during pre-specified time windows (e.g., 3, 6, 9, and 12 months) prior to the recorded date of diagnosis.

To ensure our conclusions strictly reflect our empirical data without overinterpreting the underlying timeline of symptom onset, we have revised this sentence in the Discussion section. We now frame these observations strictly around the prediagnostic period prior to cancer diagnosis. We are very grateful for this correction, which improves the methodological rigor and accuracy of our manuscript.

New text (Discussion – clean version line 401): This register-based study demonstrates that, while disparities in PHC utilization by maternal education are prevalent in the general Swedish paediatric population, these educational gradients <u>largely disappear during the 12-month period prior to childhood cancer diagnosis.</u>

9. “families from higher socioeconomic backgrounds may be more likely to bypass primary care in favour of direct specialist or emergency contact.” Again I don’t think you can infer this from the data that were analysed as you don’t have any information on specialist care.

Response: We fully agree with the reviewer and thank them for pointing out this overinterpretation. Because our dataset was restricted strictly to primary health care (PHC) registers, we do not have individual-level data on secondary specialist visits or emergency department attendances to empirically confirm whether high-education families directly bypassed primary care.

To align our discussion strictly with the data analyzed, we have removed this speculative inference from the conclusions. Instead, we now frame this solely as a potential hypothesis in the Discussion, clearly acknowledging it as an unmeasured pathway that warrants future research with linked secondary care data, while ensuring our primary conclusions remain strictly grounded in the observed PHC utilization patterns.

New text (Discussion – clean version line 406): <u>While a lower frequency of prediagnostic PHC contacts was observed among families with higher maternal education, whether this reflects a true lower reliance on health services or an alternative care-seeking pathway—such as direct contact with secondary specialist or emergency care—remains an unmeasured hypothesis. Future research integrating secondary and tertiary care registers is needed to explore these diagnostic pathways further and to inform strategies aimed at promoting equitable and timely cancer diagnosis in children.</u>

10. Figure 1 and Figure 3: x axis needs to be labelled.

Response: We thank the reviewer for pointing this out. We apologize for the oversight and have now fully updated the x-axes in both Figure 1 and Figure 3 with a more descriptive x axis label.

Furthermore, to ensure complete consistency across the manuscript and supplementary material, we have also reviewed and updated the x-axis labels across all relevant supplementary figures (Supplementary Figures 1, 3, 4, 5, and 6).

VERSION 2 – REVIEW

**REVIEWER NAME**	Harron, Katie
**REVIEWER AFFILIATION**	UCL Great Ormond Street Institute of Child Health
**REVIEWER CONFLICT OF INTEREST**	No Conflict of Interest
**AI DISCLOSURE**	No, I have not used AI
**DATE REVIEW RETURNED**	07-Sep-2026

**GENERAL COMMENTS**	The authors have responded to my previous comments and I have no further queries.
